# A Study on Light Preference in Gilts via Behavioral Pattern Analysis

**DOI:** 10.3390/ani15172620

**Published:** 2025-09-07

**Authors:** Shaojuan Ge, Haiyun Ma, Xiusong Li, Yaqiong Zeng, Baoming Li, Hao Wang, Weichao Zheng

**Affiliations:** 1 Department of Agricultural Structure and Environmental Engineering, College of Water Resources and Civil Engineering, China Agricultural University, Beijing 100083, China; geshaojuan@cau.edu.cn (S.G.);; 2 OPPLE Lighting Co., Ltd., Shanghai 201103, China; 3 Qingdao Bigherdsman (Jiaozhou) Machinery Co., Ltd., Qingdao 266300, China; 4 National Center of Technology Innovation for Pigs, Chongqing Academy of Animal Sciences, Chongqing 402460, China

**Keywords:** gilts, light color preference, behavioral analysis, precision livestock farming

## Abstract

The rational design of artificial lighting systems in pig housing can enhance animal welfare, thereby boosting gilt health and reproductive performance while improving economic metrics for swine farms. To identify the optimal light environments for gilts under artificial illumination, we conducted self-selection-based photic preference testing, ultimately providing actionable insights for welfare-centric precision lighting protocols in modern pig production. In this study, a dynamic multi-chromatic self-selection system was developed, integrating programmable RGBW-LED arrays for spectral control, inter-compartment access channels for autonomous gilt movement, and real-time image recognition technology to investigate light color preferences. Twenty-four gilts (nulliparous female pigs) were housed for five weeks in pens with white, yellow, green, blue, or red light (100 lux), and they were given free access to all of the chromatic zones through inter-compartment channels. A YOLOv8n-based deep learning framework was used to quantify their spatiotemporal distribution, activity levels, and eating behavior. The key findings were the following: (1) a significant preference for green light environments (21.29 ± 3.77% distribution proportion) (*p* < 0.05), peaking at 6:00–13:00 and 18:00–20:00; (2) the average activity was the highest in a white light environment (25.49 ± 0.77%), significantly exceeding yellow (22.69 ± 0.63%) and green light (21.55 ± 0.61%) (*p* < 0.05); and (3) the daily feed consumption under green light was the lowest, significantly lower than that under white, blue, and red light (*p* < 0.05). The findings from this study offer insights into the light environment preferences of gilts, which could improve animal welfare.

## Simple Summary

In this study, the light color preferences of gilts (nulliparous female pigs) were investigated using a self-selection experimental paradigm by granting free access to compartments illuminated with white, yellow, green, blue, or red light spectra. Their movement, activity, and feeding behavior were tracked using advanced image analysis. The key finding was that gilts showed a distinct preference for green light, particularly during the morning and evening hours. White light, in contrast, stimulated more daytime activity. Furthermore, gilts spent less time feeding in green light areas. These results reveal that gilts have innate, rhythm-based light preferences. This knowledge provides practical insights for designing lighting in pig farms that improve animal welfare and energy efficiency.

## 1. Introduction

The light environment is a critical regulatory factor in pig breeding management, as it directly impacts animal welfare, health, and productivity throughout the pig production cycle [1,2,3]. However, the industry’s rapid transition toward fully enclosed, unmanned systems has exacerbated a critical gap: most artificial lighting protocols fail to align with pigs’ visual biology and behavioral needs. This mismatch not only constrains production efficiency but may induce chronic stress, particularly in replacement gilts, in which suboptimal conditions directly jeopardize their lifetime reproductive potential. Consequently, developing precision lighting aligned with porcine ethological demands is an urgent requirement for the sustainable intensification of swine production.

With regard to light, the physiological structure and functional characteristics of animal eyes provide essential insights into visual capacity assessment [4]. Through spectral sensitivity experiments, Taylor et al. [4] determined that pigs perceive light within the 380–694 nm range, with cone cell and ganglion cell densities being approximately one-sixth of human-level densities. This unique visual architecture suggests distinct photic response mechanisms in swine. Nevertheless, most contemporary operations still employ static white light systems whose spectral composition and temporal distribution substantially mismatch pigs’ biological requirements [5]. Such misalignment not only constrains production performance but may also induce chronic stress accumulation [6,7]. Consequently, developing precision lighting protocols aligned with porcine visual characteristics and behavioral preferences represents an essential pathway toward welfare-oriented husbandry.

Behavioral modification constitutes changing animals’ primary responses to environmental stressors [8,9]. In natural conditions, animals exhibit an innate propensity to select optimal survival and reproductive environments. Thus, understanding animal requirements necessitates granting animals autonomous choice capabilities [10]. In this endeavor, reference testing emerges as the most direct and effective research paradigm for deciphering environmental needs. Recent studies demonstrate significant light preferences in pigs that directly correlate with their physiological needs. Paggi et al. [11] identified green light as being the most preferred through a comparative analysis of white, green, blue, yellow, and red spectra; Götz et al. [12] documented piglets’ initial preference for 600 lux light shifting toward darker conditions over time; and Taylor et al. [13] revealed pronounced preferences for low-intensity illumination (2.4–400 lux gradient) in young pigs. These preferences exhibit spatiotemporal dynamics—as piglets age, their exploratory preference for areas of high-intensity light decreases while their reliance on dimly lit resting areas increases [14], indicating that lighting protocols require dynamic stage-specific adjustments. Crucially, existing research predominantly focuses on piglets, with substantial knowledge gaps regarding light preferences in sows.

Due to limitations in the breeding management of multiparous sows and the estrus cycles of sows in the experimental pig farm, the health status of replacement gilts critically determines the trajectory of swine operations by directly governing the reproductive efficiency within the breeding herd. Therefore, we selected group-housed gilts as the experimental cohort for continuous investigation of their photic preferences. In order to determine the best artificial light environment for gilts, we developed a dynamic multi-chromatic self-selection system for pigs tailored to the biological characteristics of pigs. By integrating image recognition technology with deep learning algorithms, we systematically analyzed the spatiotemporal distribution patterns of gilts. Recent advances in livestock behavior monitoring, such as the real-time tracking system for cattle using modified YOLO architectures [15], demonstrate the scalability of computer vision in precision livestock farming. Building on these methodologies, we adapted the YOLOv8n network to establish a high-precision framework specifically for gilt behavior quantification under chromatic light environments. This novel approach elucidates photic preference mechanisms in intensively reared replacement gilts by analyzing spatiotemporal distribution patterns and activity rhythms under different light colors (white, yellow, green, blue, and red), ultimately advancing husbandry welfare and animal health while establishing theoretical foundations and technical protocols for light environment regulation in modern pig facilities.

## 2. Materials and Methods

### 2.1. Design of Dynamic Multi-Chromatic Self-Selection System

The system was built in a blank house of the pig nutrition and environmental regulation research base at the Chongqing Academy of Animal Sciences, and conditions pertaining to pig breeding space, breeding density, and breeding quantity were fully considered. Depending on our research goals, preference tests required the compartments to be differently arranged. Thus, we constructed five identical unit rooms in rows and performed the preference tests by setting different illumination parameters in different units.

#### 2.1.1. Design of Building Systems

**Figure 1:**
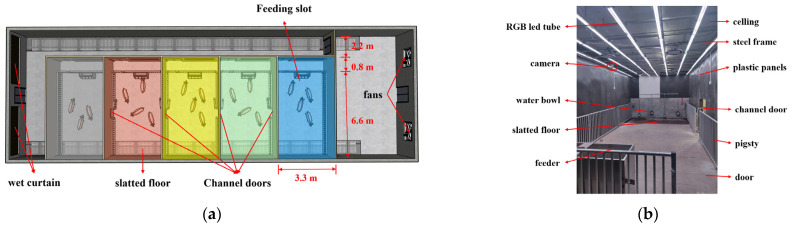
Structure of the light preference system: (**a**) schematic diagram of the swine housing units; (**b**) internal configuration of the experimental pen.

The total building area of the piggery was 24 × 9.6 × 2.7 m, and it utilized a wet curtain fan ventilation system. The house contained solid ground, with slatted floors on both sides, each with a width of 1.2 m. As part of renovation, PVC plastic panels were used to shade all the windows, and light steel keel frames and 3 mm PVC board walls were installed to divide the piggery into five independent units, each measuring 3.3 × 7.4 m (Figure 1). The PVC walls were protected by galvanized steel ring fences to prevent damage, and an operational channel space measuring 0.8 m in width was reserved at the entrance, with the actual ring fence size being 3.0 × 6.6 m. Each unit partition was equipped with a 0.8 × 1.2 m channel, allowing the pigs to pass through. The periphery of the hole was protected by a galvanized steel ring fence, and the actual width of the pig passage was 0.6 m.

The channels were shaded with a black PVC curtain. A four-hole feeding trough was installed near the aisle side, and four drinking bowls were placed above the leakage floor in order to meet the needs of the pigs in the experimental piggery. Furthermore, each unit was equipped with a door and a shutter, which served as one-way ventilation and shading for the aisle. A steel frame was built at a height of 2.4 m in the unit, which could be used to hang cameras and lamps.

#### 2.1.2. Design of Lighting Systems

Before the installation of on-site lamps, we simulated spectra, light intensity, and uniformity and determined that 98 RGB LED tubes (SH-LD02, Opple Lighting Co., Ltd., Shanghai, China) should be used in each unit to meet the requirements of illuminance and uniformity in the unit. These LED tubes were arranged in a grid pattern with equal spacing, forming seven rows and seven columns. They were closely attached in order to provide consistent illumination throughout the unit. We could change the light environment in the unit by adjusting the color and brightness of the red, green, blue, and white four-color lamp beads. To regulate the RGBW channel spectrum and brightness of the LED lamps, an 8-port digital multiplexer sub-control system (MR-218D, Beijing Mingrui Light Technology Co., Ltd., Beijing, China) was installed in each unit. This allowed for precise adjustment and synchronization of the lighting colors and intensities, ensuring a uniform light supply within the piggery. To adequately block light, the black PVC curtain was used to block the channel door between adjacent units. Additionally, the surfaces of the tubes were wiped regularly to avoid excessive dust affecting light intensity.

**Figure 2:**
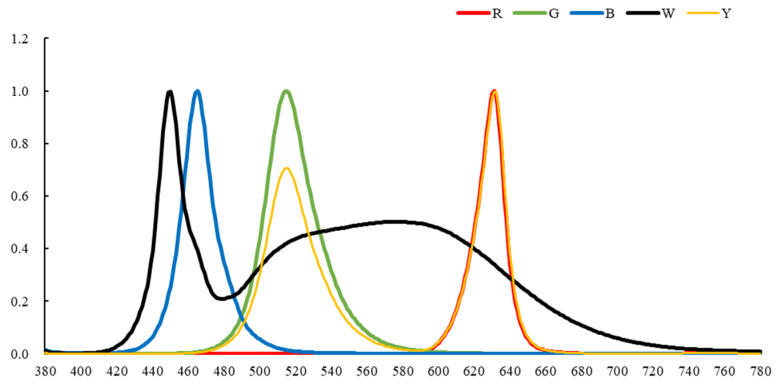
Spectral distribution of different light environments.

During the experiment, five different light environments—white light (W, spectral peak range: 420–720 nm), yellow light (Y, 500–540 nm, 620–640 nm), green light (G, 500–540 nm), blue light (B, 460–480 nm), and red light (R, 620–640 nm)—were set in the five compartments, respectively corresponding to the spectra of the different bands (Figure 2).

To ensure uniform illuminance across all rooms, a spectrometer (SRI-2000-LM, Shangze Optoelectronics Co., Ltd., Foshan, China) was used to measure and calibrate the illuminance at pig head height (approximately 0.5 m above the ground) before the formal experiment. For each room and each light color, twenty-one points (seven rows and three columns) were evenly selected for testing. The average illuminance of these test points was calculated and used as the basis for adjusting the numerical parameters of the controller to bring the indoor lighting close to the target value of 100 lux. Throughout the experiment, the light phase took place from 5:00 to 21:00. The illuminance in each compartment was continuously maintained at 100 ± 2 lux for all light colors, which was verified by weekly spot-checks with the same spectrometer and adjustments.

### 2.2. Experimental Design

The experiment was carried out from May 2022 to July 2022. During the experiment, the ambient temperature was 20~31 °C, and the relative humidity was 55~95%.

A total of 30 5-month-old gilts (Large White × Landrace) of similar age and weight were selected from the same breeding house, with the average weight of these being 81.40 ± 10.54 kg. At the end of the experiment, the average weight of gilts was 116.79 ± 12.43 kg (5 days of pre-experiment, 35 days of formal experiment, a total of 40 days). On the first day of the pre-experiment, the 30 gilts were randomly divided into five groups, and 6 pigs in each group were distributed in different units to ensure even distribution in the initial state of the experiment. A 5-day pre-experiment was conducted to alleviate the stress caused by the environmental replacement. During the pre-experimental period, all channel doors were opened in order to train the pigs to use them, with the pigs being able to move freely among all the compartments. Additionally, according to the lighting schedule in Table 1, the internal lighting environment was changed appropriately every day to potentially eliminate other factors that could affect the pigs’ preferential choices.

Our formal experiment spanned 35 days, with every 7 days designated as an experimental cycle. Additionally, the lighting scheme was adjusted once per cycle in the test compartment (Table 1) to prevent errors caused by environmental fluctuations or individual pig position preferences. Within each illumination scheme, the light color order and compartment order were randomized by using a Latin square table in order to eliminate positional influences on the gilts, such as gilt preference for fixed positions, during the experiment. Moreover, during the experiment, the pigs were free to feed and drink water. The remaining feed from the day was removed during 9:00–10:00 a.m. every day with the use of an industrial vacuum cleaner, and the pigs’ weight was recorded. Then, an appropriate amount of clean feed was added via an automatic feeder. The piggery was cleaned every day during 9:00–10:00 a.m. and 15:00–16:00 p.m.

### 2.3. Data Collection and Image Processing

#### 2.3.1. Data Collection

Five wide-angle cameras (3346P1-1, Hangzhou Hikvision Digital Technology Co., Ltd., Hangzhou, China) installed at a height of 2.4 m from the ground and a network video recorder (7808N-K1/8P, Hangzhou Hikvision Digital Technology Co., Ltd., Hangzhou, China) were used to record each test unit for 24 h, and the daily lighting period ranged from 05:00 to 21:00. Images were systematically captured at 1-min intervals per unit to prevent overrepresentation, yielding a dataset of 5750 frames for active behavior modeling and 1660 frames for feeding behavior analysis. To control for spectral variation effects on the recognition accuracy, samples were near-uniformly distributed across all experimental light colors (white/yellow/green/blue/red). The image pixels were 1920 × 1080 in the model dataset. Because the light color in the images could interfere with recognition accuracy, image saturation was reduced to 10% in advance, and the dataset was then collected and processed. The model images used were at least one minute apart, thus reducing similar images and increasing the model’s accuracy.

#### 2.3.2. Model Training and Behavior Definition

For automated detection and classification of pig behaviors, we employed the YOLOv8 (You Only Look Once version 8) algorithm, specifically the nano variant (YOLOv8n), which provides an optimal balance of speed and accuracy for real-time applications. In this aim, an accurate and fast pig counting and conventional posture recognition model based on the YOLO series algorithm was constructed via the YOLOv8n network structure. Similar frameworks have been deployed for cattle tracking [15].

To obtain position and posture information from the model, data must be labeled. Therefore, the images in this study were labeled with the use of the graphical annotation website https://www.makesense.ai/ (accessed on 20 July 2022), and the format required by YOLO was saved as a label file. Four kinds of labels were configured—standing posture, lying posture, sitting posture, and eating posture—and the definitions of these behaviors are shown in Table 2. Activity levels were quantified as the proportion of time spent in active states (standing or sitting) versus inactive states (lying). After marking each image, a txt file with the same name was generated, and the pose category of the target pig in the image and the center coordinates, height, and width of the mark box were recorded. The labeled dataset was divided into a training set and a validation set according to a ratio of 8:2 for model training.

**Figure 3:**
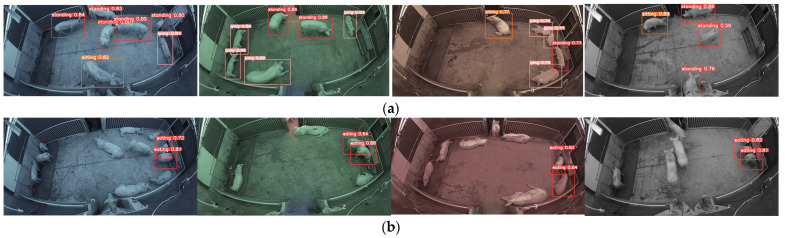
Recognition after image color saturation was reduced to 10%, where (**a**) shows the results of lying, standing, and sitting behavior recognition under different light colors (pink, red, and orange recognition boxes, respectively); (**b**) shows the results of eating behavior recognition under different light colors.

The behavior recognition model was developed on a Windows 11 64-bit operating system utilizing the PyTorch3.10 framework with CUDA11.1 acceleration, powered by an NVIDIA GeForce GTX4070Ti GPU (12GB VRAM) and 32GB RAM. Leveraging the YOLOv8n.pt pre-trained model with a batch size of 64 over 225 training epochs. Considering the problem of repeated recognition between active behavior and eating behavior, we trained two different recognition models. As shown in Figure 3, one is an active/inactive behavior recognition model (including lying, standing and sitting behaviors), and the other is a separate eating behavior recognition model. The system achieved recognition accuracies exceeding 90% thresholds for four key porcine behaviors—standing (97.5%), sitting (90.3%), lying (98.5%), and eating (95.2%)—meeting the accuracy requirements for pig behavior recognition. Misidentification primarily occurred during huddled resting periods, where overlapping bodies complicated detection. Additionally, the recognition of sitting postures demonstrated a lower accuracy due to this transient stance occurring infrequently, and predominantly during environmental observation, resulting in limited training data for this posture.

#### 2.3.3. Duplicate Count Correction

Image analysis revealed that pigs in channel door areas between the compartments were captured by adjacent cameras simultaneously, leading to duplicate counts exceeding the actual population. To resolve this, we predefined monitoring zone boundaries using pixel coordinates (x, y). By detecting real-time pig coordinates and analyzing their spatial relationships with predefined zones, the system automatically filtered data to only retain individuals appearing in unilateral access areas, effectively eliminating cross-zone counting errors. The finalized outputs included zone codes, pig categories, and spatial coordinates, guaranteeing spatiotemporal uniqueness for each individual.

### 2.4. Calculation of Indices

The key indices analyzed in this study—activity level, feeding time, and feed consumption—were calculated as follows:

(1) Activity Level was defined as the proportion of gilts in active states (standing or sitting) relative to the total identified population across all five units at each sampling point. It was calculated for every captured image frame using the formula: Activity Level (%) = (Number of active gilts/Total number of detected gilts) × 100%. The average values for specific time periods were then derived.
(2) Feeding Time per gilt per day was calculated based on the frequency of the “eating” behavior detected by the model. Given the image sampling interval of 1 min, the total daily feeding time (in minutes) for an individual was estimated as: Feeding Time (min) = (Number of frames labeled as ‘eating’ all of the days)/30 gilts. The results are presented as the mean feeding time per gilt within each light-color environment.
(3) Feed Consumption was measured gravimetrically for each chromatic unit. The daily consumption was calculated by subtracting the weight of the residual feed collected at 09:00 the following morning from the weight of the fresh feed provided at 09:00 each day. An industrial vacuum cleaner (YZ206A, China Yangzi Group, Chuzhou, China) was used to suck out the remaining feed. To account for the group-housing design, the distribution proportion of daily feed consumption for each unit was used for statistical analysis, calculated as: Consumption Proportion of Unit (%) = (Daily consumption of Unit/Total daily consumption of all units) × 100%.

The resulting data from these calculations were then subjected to the statistical analysis described in Section 2.5.

### 2.5. Statistical Analysis

In this study, the data were analyzed and calculated using Microsoft Excel 2019 (Microsoft Corporation, Microsoft Corporation, Redmond, WA, USA), and charts were made using Origin 2021 (OriginLab Corporation, Northampton, MA, USA). IBM SPSS Statistics 22.0 (IBM SPSS statistics for Windows, version 22.0. IBM Corp, Armonk, New York, USA) was used for linear mixed model analysis to exclude the interference of unit position and test time. The model used light intensity, unit position, and their interactions as fixed effects, and it used the interactions of test time, light intensity, and unit position as random effects. The following mixed model was established with *p* < 0.05 as the significance level:

*Y_ijk_* = *μ* + *I_i_* + *S_j_* + (*IS*)_*ij*_ + *T_k_* + (*IST*)_*ijk*_ + *ε_ijk_*

In this model, the dependent variable (*Y_ijk_*) represented one of two primary outcome measures: (1) the daily spatial distribution proportion, calculated as the number of gilts in unit *j* under light color *i* on day *k*-th divided by the total number of gilts across all five units, or (2) the proportion of gilts within unit *j* exhibiting a specific behavioral state (active or inactive) on day *k*. The term μ represents the overall mean of the dependent variable. The fixed effects were defined as follows: *I_i_* denotes the light color treatment (a categorical variable with five levels: white, yellow, green, blue, or red, all at 100 lux), *S_j_* represents the positional effect of the physical unit (Unit 1 to Unit 5), and *(IS)_ij_* is their interaction term. The random effects included *T_k_*, accounting for variability across test days, and (*IST*)*_ijk_*, which models the complex variance associated with each unique light-color-position combination across days, with ε_ijk_ representing the residual random error.

## 3. Results

### 3.1. Temporal and Spatial Preference for Light Color

**Figure 4:**
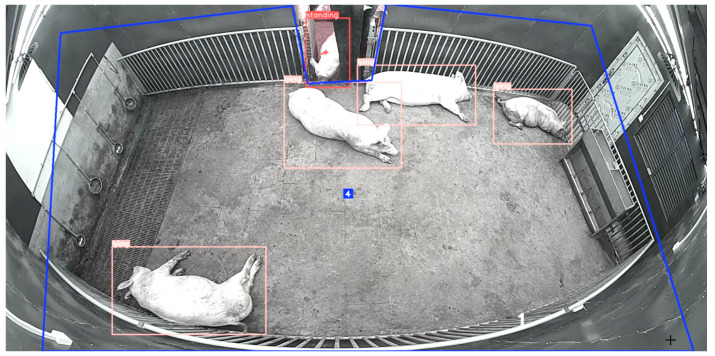
Identifiable zones for behavioral recognition. The blue box in the figure represents the identifiable area. The 4 in the middle of the image indicates that there are four pigs in the recognition area.

**Figure 5:**
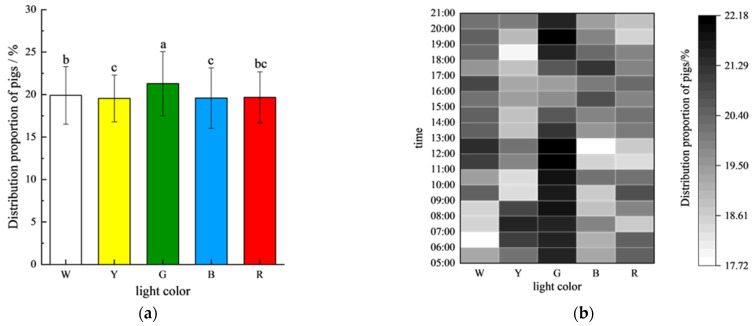
Pig distribution proportions under different light colors. (**a**) Full-time average distribution. (**b**) Time-of-day specific distribution. The different lowercase letters (a, b, and c) indicate significant differences between different testing stages, *p* < 0.05, and the same letter indicates no significant differences.

Figure 4 illustrates the distribution of the pigs across different periods under various colored light environments during the experiment. Overall, the gilts exhibited a relatively uniform distribution across the five light color environments (Figure 5a). However, a significant preference for the green light environment (G: 21.29 ± 3.77%) was observed compared to all other colors (*p* < 0.05). The distribution under white light (W: 19.91 ± 3.38%) was significantly higher than that under yellow (Y: 19.54 ± 2.75%) and blue light (B: 19.58 ± 3.56%) (*p* < 0.05), but not different from that under red light (R: 19.68 ± 3.00%). No significant differences were found between the red, yellow, and blue light environments (*p* > 0.05) (Figure 5a).

The analysis of the temporal patterns revealed distinct diurnal preferences (Figure 5b): the gilts consistently showed the highest occupancy in the green light environments during the 16-h photoperiod, and this was significantly higher than that under white light from 06:00–07:00 and 08:00–09:00; yellow light from 09:00–11:00 and 18:00–19:00; blue light from 08:00–10:00 and 11:00–13:00; and red light from 11:00–13:00 to 19:00–20:00 (all *p* < 0.05). Notably, this clear chromatic hierarchy was absent during the afternoon (13:00–18:00), with no significant differences in distribution observed between the colors (*p* > 0.05), suggesting circadian modulation of light color preferences.

### 3.2. Activity Behavior of Pigs Under Different Light Colors

During the experiment, the average activity levels of the gilts in the five light color environments ranged from 21% to 26%, and during the lighting period, the average activity level of the pigs in the piggery was 23.67 ± 2.14%. The highest average activity level was observed under white light (W: 25.49 ± 0.77%), significantly exceeding the levels seen under yellow (Y: 22.69 ± 0.63%) and green light (G: 21.55 ± 0.61%) (*p* < 0.05). The activity under blue (B: 24.86 ± 0.38%) and red light (R: 24.34 ± 0.64%) was intermediate and not significantly different from that under white light, but was significantly higher than that under green light (*p* < 0.05).

**Figure 6:**
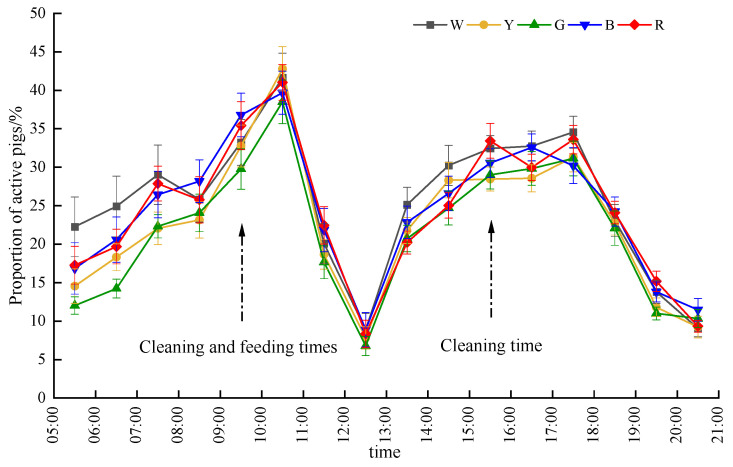
Hourly activity levels across light color environments. The abscissa 05:30 time from 05:00 to 06:00 in the diagram represents the average value in an hour during the test period.

The proportion of active pigs under different light color environments at various time points is shown in Figure 6. The activity level of the pig herd was influenced by the lighting time, gradually increasing after the lights were turned on and reaching a peak during 10:00 to 11:00 am, which can be attributed to the gilts’ activity habits and human interventions. The activity level rapidly decreased from 11:00 to 12:00 am after the experimenters and caretakers left. A collective rest period occurred around noon, with over 90% of the pigs lying down, leading to a decline in activity level. Subsequently, it gradually increased again.

Significant differences were only observed under white light and green light during the first two hours after the lights were turned on (*p* < 0.05), with significantly higher activity levels in the white light group compared to the green light group (*p* < 0.05). Between 19:00 and 20:00, the activity level under red light was significantly higher than that under green light (*p* < 0.05). In the other periods of the experiment, no significant differences in activity levels were found among the pigs under different light color environments.

**Figure 7:**
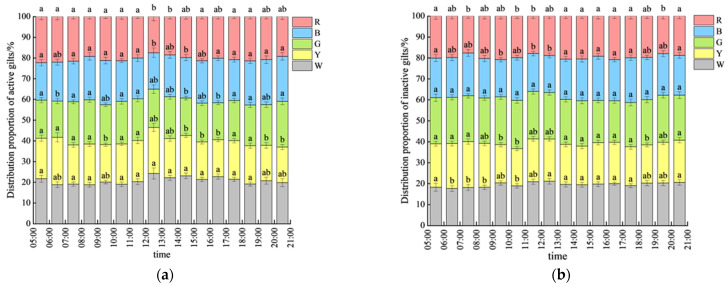
Spatial distribution of active and inactive pigs. (**a**) Active state distribution. (**b**) Inactive state distribution. The different lowercase letters (a, b) within the same column indicate significant differences between different testing stages, *p* < 0.05.

Figure 7 illustrates the proportional distribution of the pigs in active and inactive states under different light colors. During the light period, the average distribution proportions of active pigs under the five light colors were as follows: white light (W), 20.81 ± 1.34%; yellow light (Y), 19.17 ± 1.27%; green light (G), 19.48 ± 1.23%; blue light (B), 20.12 ± 1.47%; and red light (R), 20.42 ± 1.23%. The distribution of active pigs under white light consistently remained at a higher level, while the other four light colors showed significantly lower distributions compared to that under white light (*p* < 0.05). Overall, the distribution of pigs under different light environments during the light period was relatively uniform, with the most significant differences observed between 12:00 and 13:00, which may be related to the lowest number of active pigs during this period.

During the light period, the average distribution proportions of inactive pigs under the five light colors were as follows: white light (W), 19.56 ± 1.21%; yellow light (Y), 19.72 ± 1.12%; green light (G), 21.74 ± 1.32%; blue light (B), 19.47 ± 1.29%; and red light (R), 19.51 ± 1.20%. Because inactive pigs accounted for more than 76% of the total population throughout the experimental phase, the distribution of inactive pigs followed a similar trend to the distribution overall. The number of lying pigs under green light was significantly higher than that under other light colors between 6:00–13:00 and 18:00–20:00 (*p* < 0.05), whereas no significant differences were observed among the groups during other time periods (*p* > 0.05).

### 3.3. Eating Behavior of Pigs Under Different Light Colors

**Figure 8:**
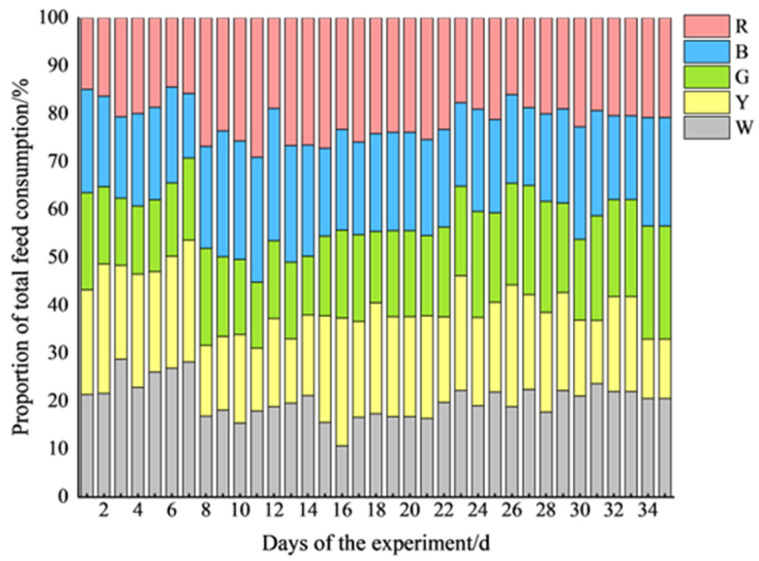
Total feed consumption proportions under different light colors.

Figure 8 demonstrates the daily feed consumption of pigs under different light conditions during the experiment. Overall, the feed consumption fluctuated significantly in the first two weeks, with notable differences observed between all of the groups. During the first experimental phase, the feed consumption distribution showed significant fluctuations (Days 7–8, corresponding to the first light color adjustment), with higher feed intake under white and yellow light compared to that under green, blue, and red light (*p* < 0.05) (Figure 8). The consumption rate stabilized after approximately two weeks, becoming more uniform across the different light colors. Throughout the experimental period, the feed consumption proportions for each group were as follows: white light (W), 20.22 ± 3.74%; yellow light (Y), 19.63 ± 3.97%; green light (G), 18.10 ± 2.96%; blue light (B), 20.59 ± 2.96%; and red light (R), 21.45 ± 3.77%. Among these, the daily feed consumption under green light was the lowest, significantly lower than that under white, blue, and red light (*p* < 0.05), whereas the daily feed consumption under red light was significantly higher than that under yellow light (*p* < 0.05).

**Figure 9:**
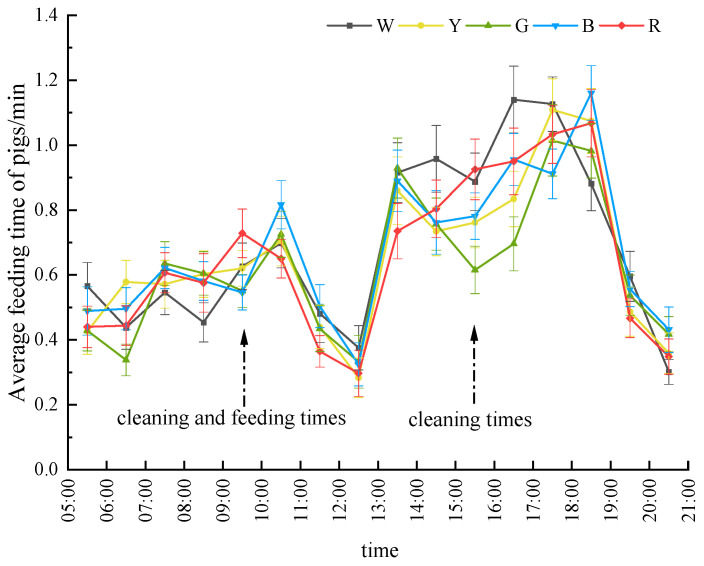
Feeding time allocation choices across light color environments.

Figure 9 presents the average feeding time of gilts during the light period in the experiment, demonstrating that the feeding time of gilts under white light was significantly higher compared to that under yellow and green light environments (*p* < 0.05). Similarly, the average feeding time under red light was significantly higher than that under green light (*p* < 0.05). However, no significant differences in feeding frequency were observed among the other groups. During the experimental stage, the pigs exhibited a concentrated feeding pattern in the afternoon, with feeding time from 13:00 to 18:00 accounting for more than 50% of the total feeding time.

Significant variations in feeding time were noted at specific time intervals. From 6:00 to 7:00, the feeding time under green light was significantly lower than that under yellow light (*p* < 0.05); from 9:00 to 10:00, the average feeding time under green and blue light was significantly lower than that under red light (*p* < 0.05). In addition, from 15:00 to 16:00, green light environments exhibited significantly shorter feeding durations compared to white and red light (*p* < 0.05); during 16:00–17:00, both yellow and green light conditions showed significantly reduced feeding times relative to white and blue light (*p* < 0.05), whereas at 18:00–19:00, white light was associated with the lowest feeding engagement, being statistically lower than blue light (*p* < 0.05). No significant differences were observed among the groups during other time periods (*p* > 0.05). These temporally stratified patterns demonstrate a light color-dependent modulation of feeding rhythms, with chromatic efficacy varying across diurnal phases.

## 4. Discussion

Through the dynamic multi-chromatic self-selection system, this study represents pioneering research on the systematic elucidation of light color preferences and circadian characteristics in gilts under intensive farming conditions. The effects of light color across three behavioral dimensions—spatial distribution, activity intensity, and feeding patterns—are quantified, demonstrating that respecting circadian-driven light color preferences significantly enhances gilt welfare and production efficiency in intensive systems.

### 4.1. Distribution Patterns: Light Color Choices and Circadian Rhythms

Through a spatiotemporal distribution analysis performed under different light color conditions, it was found that gilts exhibit a significant preference for green light environments. Given that the spatial distribution patterns of freely moving animals can directly reflect their environmental preferences and needs [16,17], the experimental design in this study ensured equal resource conditions (sufficient food, water, and activity space) in each unit to minimize interference from social competition. Additionally, stocking density was maintained within normal limits (each pen accommodated approximately 50% of the test pigs) to guarantee unrestricted movement. The results showed a relatively uniform distribution across light color environments (maximum difference <5%), which may be related to the dispersed behavior of pigs during the summer. Notably, the proportion of pigs under green light was significantly higher than under other color environments (*p* < 0.05), with pronounced preference peaks observed between 6:00–13:00 and 18:00–20:00. These findings align with those of Paggi et al. [11], who reported similar green light preference in weaned piglets.

From a biological perspective, pigs’ retinal cone cells exhibit peak sensitivity at 439 nm (blue light) and 556 nm (green light) wavelengths [18], which may make green light environments appear brighter or more comfortable, thereby driving the observed preference. Furthermore, Taylor et al. [4] noted that wild boars in natural habitats preferentially select environments with green foliage coverage. This suggests an evolved ethological attraction to green spectra, which likely signifies shelter, safety, and abundant resources, thereby reducing stress and promoting resting behavior. This mechanism aligns with our findings of increased inactive behavior (lying) in green zones. The significant morning and evening preference for green light (*p* < 0.05) observed in this study may also relate to the circadian rhythms and natural foraging patterns of pigs. These time windows coincide with critical transitions in natural light intensity, where green light may better align with their biological requirements.

Compared to conventional static light environment paradigms, the dynamic spatial selection mechanism better aligns with swine ethological needs, and the integration of YOLOv8-based high-precision behavior recognition successfully quantifies the effects of light color across three behavioral dimensions: spatial distribution, activity intensity, and feeding patterns. Although previous studies have confirmed the proclivity of piglets for green light [11], our findings reveal an enhanced preference for green light in gilts at dawn/dusk and the diurnal activity-modulating specificity of white light, suggesting developmental divergence in photic response mechanisms across growth stages

### 4.2. Activity Levels: Light Color Effects on Movement

This experiment revealed that, under constant 100 lux illumination, the activity levels of pigs exhibited bimodal fluctuations after light activation, with a steeper rise in the morning and a gentler afternoon variation. The light color showed no significant impact on active state proportions during most of the periods (*p* > 0.05), except for specific intervals such as the activity trough between 12:00 and 13:00. These findings align with those of Svenga et al. [3], who documented increased brief lying behavior during noon periods in pigs. Notably, the observed pattern of activity decline and trough visitation at 12:00–13:00, followed by gradual recovery, was also replicated in our previous study on light intensity preferences in gilts [19]. These collective findings suggest that incorporating intermittent dark phases (e.g., introducing short dark periods at noon) in full-day lighting systems may better align with porcine circadian rhythms while achieving energy-saving benefits.

Additionally, the preference rhythm (peak during dawn and dusk) may be linked to circadian regulation. Green light has sedative effects in other species and may affect melatonin and thyroid hormone signaling pathways by inhibiting the expression of Mtnr1 b and Thrβ receptors, unlike the stimulating effect of brighter white light [20]. This could explain the temporal shift in preference and the higher activity levels we recorded under white light.

Existing research indicates that conventionally raised pigs spend 80–90% of their daily time in inactive states [21]. In this study, the proportion of inactive pigs was 76.33 ± 2.14%, lower than full-day averages due to extended nighttime rest periods. In their color temperature preference study, Götz et al. [22] proposed the theory that pigs preferentially select comfortable resting areas while avoiding waste elimination in these zones. This behavioral mechanism may explain the spatial stability of the inactive pig distribution observed in our experiment; once optimal resting locations are established, positional changes become infrequent. Future investigations integrating elimination behavior monitoring could further elucidate the comprehensive mechanisms underlying light color effects on spatial selection behavior.

### 4.3. Feeding Patterns: Light Colors with Feed Consumption and Feeding Time

During days 7–8 of the experiment, when the light color positions were first adjusted, the feed intake distribution of gilts across different light compartments changed significantly. This likely resulted from environmental stress caused by abrupt light color changes, prompting the pigs to maintain their original feeding positions. Over time, the pigs gradually adapted to the modified light environment (with reduced feed intake fluctuations and diminished intergroup differences after approximately two weeks), indicating the necessity of adequate acclimation periods for lighting environment modifications. Notably, white and yellow light environments demonstrated superior feeding stability compared to other colors, likely due to their spectral similarity to the natural light conditions prior to relocation. This aligns with a study by Tanida et al. [23], who proposed “behavioral inertia towards original rearing environments” in animals. Frase et al. [24] further cautioned that animal preferences may be conflated with environmental familiarity, which explains the differential responses observed in this study—the delayed adaptation to green light versus the immediate aversion to blue light.

In this study, video sampling at one frame per minute was employed and combined with image recognition to track trough visitation frequency as a predictor of light color preferences. The recorded daily feeding duration during lighting phases (52.7 ± 1.31 min) closely matched the standard range for 80–100 kg pigs (49.6–65.1 min) [25], confirming the representativeness of lighting-phase data. However, the aversion to blue–green-colored feed reported by Taylor [4] was not replicated here, possibly due to fundamental design differences, given that this study focused on global light environment adaptation, whereas Taylor’s experiment involved localized trough lighting. This discrepancy suggests that porcine light color preferences exhibit spatial scale dependency; behavioral responses to whole-unit light environments may fundamentally differ from those to localized color stimuli. Future studies should implement zonal lighting designs to systematically dissect the spatial regulation mechanisms of light environments on feeding behavior.

## 5. Conclusions

In this study, we systematically revealed the light color preference patterns of gilts under intensive farming conditions through a multi-chromatic, dynamic multi-chromatic self-selection system for pigs integrated with high-precision image recognition technology. The results demonstrated that green light environments significantly increased the pig distribution numbers in the morning (6:00–13:00) and evening (18:00–21:00) periods (*p* < 0.05), whereas white light elevated the daytime activity peaks by 25.49 ± 0.77%. The feeding behavior analysis performed further indicated shorter daily feeding durations under green light compared to white light, with red light exhibiting stable feeding performance throughout the day. These findings validate the phase-specific regulatory effects of light color parameters on swine behavior and provide critical data for optimizing intelligent light environment designs in modern pig housing systems.

Future research should explore the mechanistic links between light color parameters and sow reproductive performance while extending this model to validate its applicability across different pig breeds and growth stages. Guided by animal welfare principles, these discoveries provide novel insights into porcine visual behavioral ecology while offering strategic implications for redefining light environment management protocols in commercial pig production systems.

## Figures and Tables

**Table 1:** Phase-specific light color allocation in the experimental units.

Lighting Scheme	Light Color				
	Unit 1	Unit 2	Unit 3	Unit 4	Unit 5
Plan 1	W	Y	G	B	R
Plan 2	R	B	W	Y	G
Plan 3	Y	R	B	G	W
Plan 4	G	W	Y	R	B
Plan 5	B	G	R	W	Y

In the table, W, Y, G, B, and R represents white, yellow, green, blue, and red light colors.

**Table 2:** Definitions of the pig behaviors measured.

Behaviors	Definition
Active behavior	
Standing	The body is supported by limbs to stand.
Sitting	The body is supported by one or two forelimbs.
Inactive behavior	
Lying	The body is close to the ground, with the limbs unfolded or under the body.
Eating	The pig’s head is down and coincides with the feeding trough.

## Data Availability

Data will be made available on reasonable request from the corresponding author.
